# Unaltered TNF-α production by macrophages and monocytes in diet-induced obesity in the rat

**DOI:** 10.1186/1476-9255-2-2

**Published:** 2005-03-21

**Authors:** Sammy Bedoui, Elena Velkoska, Steve Bozinovski, Jessica E Jones, Gary P Anderson, Margaret J Morris

**Affiliations:** 1 Department of Pharmacology, The University of Melbourne, Melbourne, 3010, Australia; 2 Department of Medicine, The University of Melbourne, Melbourne, 3010, Australia; 3 Cooperative Research Centre for Chronic Inflammatory Diseases, The University of Melbourne, Melbourne, 3010, Australia

**Keywords:** innate immunity, leptin, lipopolysaccharide, macrophage, neuropeptide Y, obesity, tumour necrosis factor

## Abstract

**Background:**

Recent findings have established an association between obesity and immune dysfunction. However, most of the studies investigating the effects of obesity on immune function have been carried out in genetically obese rodent models. Since human obesity is mostly due to intake of a high fat diet and decreased energy expenditure, we asked whether immunological defects also occur in diet-induced obesity. Specifically, we focused on the function of monocytes and macrophages, as these cells are thought to be involved in the low-grade inflammation present in obesity.

**Methods:**

Male Sprague-Dawley rats were fed a high-fat or a standard chow diet for either 2 or 10 weeks. At the end of the intervention period animals were anaesthetised, blood collected for determination of plasma mediator concentrations and lipopolysaccharide (LPS) stimulated production of TNF-α by monocytes. LPS stimulated production of TNF-α in alveolar macrophages was also determined.

**Results:**

High-fat feeding for either 2 or 10 weeks resulted in significant increases in fat mass and serum leptin. Although increased serum leptin has previously been linked to modulation of innate immunity, we found no significant difference in the LPS stimulated production of TNF-α by either blood monocytes or alveolar macrophages between the dietary groups. Furthermore, we failed to find a significant increase in circulating TNF-α concentrations in obese animals, as reported for genetically obese animals.

**Conclusion:**

Our data suggest that defects in innate immune function observed in genetically obese animals are not mimicked by dietary obesity, and may more likely reflect the gross abnormality in leptin function of these models. Further work is required delineate the effects of dietary obesity on inflammatory state and immune function.

## Background

Obesity is a very common chronic disease that poses significant health risks such as diabetes, cardiovascular diseases and hypertension. This pathological condition is characterized by complex neuroendocrine changes in the brain as well as in the periphery, involving mediators such as neuropeptide Y (NPY) and leptin [1]. Additionally, there have been several reports demonstrating that obesity is associated with altered immune function and a chronic low-grade inflammatory status [summarized by [2,3]]. Specifically it has been reported that obese individuals have a higher incidence and severity of infectious diseases [4]. These defects also include disturbances in macrophage mediated phagocytosis and pro-inflammatory cytokine production [5] as well as increased sensitivity to endotoxin-induced lethality [6]. To date, experimental approaches to the investigation of this novel link between obesity and immune function have been predominantly carried out in genetic models of obesity that either lack leptin (*ob/ob *mouse) or the long form of the leptin receptor (*db/db *mouse). However, leptin mutations only account for a small fraction of obesity in humans with the majority of obesity linked to overnutrition and reduced energy expenditure [7]. With leptin resembling several aspects of a cytokine and exerting various immunological functions [reviewed by [8]], it is unclear whether these models examine the effects of obesity in general or rather the effects of a defective leptin system on immune function. This question needs to be addressed by investigating immune function in diet-induced models of obesity.

Monocytes and macrophages are major cellular components of the innate branch of the immune system. With their ability to produce cytokines, e.g. tumour necrosis factor-α (TNF-α), in response to bacteria and bacterial fragments, such as LPS, monocytes and macrophages are essential to the first line of defence at contact sites between the interior and the exterior, such as the mucosa of the lungs or the gastrointestinal tract. Notably, increasing evidence suggests that macrophages also play an important role in the development of the low-grade inflammation that is present in obesity. Recent work demonstrates that macrophages infiltrate the adipose tissue and that these cells are integral to the low grade-inflammation [9]. However, many questions remain unanswered regarding the precise role of monocytes and macrophages in the course of obesity. For example, it is of great interest to examine whether the functional changes described within the adipose tissue are intrinsic to the macrophages, or whether these defects result from the interaction with the local microenvironment in the adipose tissue. If intrinsic macrophage defects are responsible for the described alterations in obesity, similar defects should also be present in other macrophage compartments. Therefore, the aim of the present study was to investigate macrophage and monocyte function in compartments other than the adipose tissue of obese animals, specifically the lungs and the blood.

In order to examine monocyte and macrophage function in diet-induced obesity, we subjected male Sprague Dawley rats to a cafeteria-style diet lasting either 2 weeks (short term) or 10 weeks (long term). In this way we could examine the effects of diet *per se *and of established obesity. Litter mates received normal rodent chow diet. Upon completion of the dietary intervention, blood monocytes and alveolar macrophages were collected and stimulated with LPS *in vitro*. Under these conditions LPS induces strong production of the pro-inflammatory mediator TNF-α [10]. As leptin and sympathetic activation impact on immune function [11,12], plasma concentrations of NPY, a marker for sympathetic nervous system activity [13,14], and leptin, as well as TNF-α were also determined.

## Materials and methods

### Animals

Male Sprague-Dawley rats were kept under controlled light (06.00–18.00 h) and temperature (20 ± 2°C) conditions with *ad libitum *access to water. Five week old rats (n = 18) were randomly divided into two groups. The control group ("controls", n = 9) was fed standard laboratory chow (12.5% calories as fat) and the second group ("high-fat diet", n = 9) was presented with a highly-palatable high-fat cafeteria-style diet (35% calories as fat), consisting of meat and pastry pies, pasta and cake and supplemented chow. Two different sets of experiments were conducted. The first series ("long term diet") was maintained for 10 weeks, whereas a second series ("short term diet") was only fed for 2 weeks. Both experimental sets consisted of control animals and diet-induced obese animals that were assigned to groups of similar starting weights. Body weight and caloric intake of all rats was monitored weekly. All procedures were approved by the Animal Experimentation Ethics Committee of the University of Melbourne.

### Collection of tissues

At the completion of the dietary period, the animals were anaesthetized with pentobarbital (Nembutal, 100 mg/kg, Merial Australia Pty Ltd, Australia). Cardiac puncture (3 ml) was performed using a heparinised syringe to collect blood for full blood stimulation, and to allow preparation of plasma for determination of plasma mediator concentrations. Retroperitoneal white adipose tissues and the spleen were removed and weighed.

### Bronchoalveolar lavage

To obtain alveolar macrophages, anaesthetized rats were subjected to bronchoalveolar lavage (BAL). The lungs were rinsed with 10 ml of cold, sterile PBS via a cannula placed into the trachea. The lungs were washed twice and total cell counts and viabilities were determined by ethidium bromide/acridine orange (Molecular Probes, Oregon, USA) fluorescent viability stains using a Neubauer hemocytometer. Cytocentrifuge preparations (Shandon Cytospin 3) using 100 μl of BAL were differentiated according to standard morphological criteria counting at least 500 cells (DiffQuik, Zeiss, Germany). BAL fluid contained between 97–99% alveolar macrophages. Alveolar macrophages were adjusted to 500,000 cells/250 μl and stimulated for 3 h at 37°C in the presence of various concentrations of LPS (0.001–10 μg/ml, *E. Coli *Serotype 026:B6, Sigma). Supernatants were collected and stored at -80°C for measurement of TNF-α.

### Full Blood stimulation

Various concentrations of LPS (0.01–10 μg/ml) were added to 250 μl full blood and incubated for 3 h at 37°C. Upon completion of the incubation, samples were centrifuged and supernatants were stored at -80°C for measurement of TNF-α.

### Detection of TNF-α, NPY and Leptin

All reagents were endotoxin-free to ensure that TNF-α was not artifactually induced, except where LPS was deliberately used. The concentration of TNF-α in the supernatants and plasma samples was determined by a commercially available ELISA kit (Pharmingen, Merckville, Australia) with standard concentrations ranging from 4–1000 pg/ml. Plasma leptin concentrations were measured using a commercially available radioimmunoassay kit (Linco, Missouri, USA) while NPY was measured using an in house assay utilising a rabbit antibody and ^125^I-NPY (2000 Ci/mmol, Amersham, Australia) as previously described [13].

### Statistics

Student's unpaired t-test was used to determine significant differences for organ masses and concentrations of NPY, leptin and TNF-α. Data from BAL and full blood stimulation was analysed using one-way ANOVA and body weight data was subjected to ANOVA for repeated measures with subsequent LSD if p-values were below p < 0.05. Differences where p-values were <0.05 are considered significant. Statistics were performed using GraphPadPrism 3.0 for Windows.

## Results

### Effect of high-fat diet on caloric intake, body weight and organ mass

**Figure 1:**
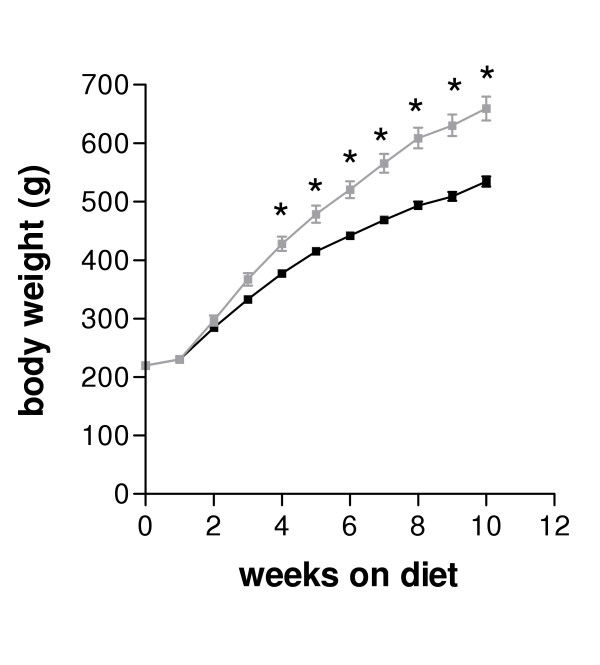
Body weight (g) of diet-induced obese (grey squares) and control (black squares) Sprague-Dawley rats following exposure to a cafeteria-style high fat diet or standard laboratory chow. Results are expressed as mean ± SEM (n = 9 diet-induced obese rats, n = 9 control rats). Data were analysed by ANOVA for repeated measures and significant differences (p < 0.05) are indicated by asterisks.

Exposure of animals to the high-fat diet led to significant increases in caloric intake (p < 0.05; Table 1) and body weight from 3 weeks (p < 0.05; Fig. 1). Animals on the high-fat diet continued to gain weight and at the completion of the 10 week dietary intervention weighed 23% more than their respective controls. Even though body weight was not different, retroperitoneal white adipose tissue was already significantly increased after 2 weeks on the diet (p < 0.05; Table. 1). Continued exposure to the high-fat diet lead to progressive increases in caloric intake, and adipose tissue mass, which was 2.8 fold higher than the control animals at 10 weeks of diet (Table 1). Net spleen weight was significantly depressed (p < 0.05) after 2 weeks of high-fat diet. Although there was a tendency for reduced spleen mass after 10 weeks of dietary intervention, this did not reach statistical significance (Table 1).

**Table 1:** Parameters of the model of diet-induced obesity.

	**Short term diet (2 weeks)**		**Long term diet (10 weeks)**	
	
	**Chow**	**High Fat**	**Chow**	**High Fat**
Caloric intake (cal/day)	95.6 ± 3.0	178.6 ± 19.1*	97.6 ± 11.3	229.9 ± 8.9*
Body weight (g)	287.5 ± 2.2	302.3 ± 5.0	515.6 ± 9.1	635.3 ± 12.3
White adipose tissue (g)	1.4 ± 0.1	2.7 ± 0.2 *	4.5 ± 0.4	12.4 ± 1.5*
Spleen (g)	0.88 ± 0.03	0.79 ± 0.02*	0.97 ± 0.04	0.89 ± 0.05
TNF-α (pg/ml)	5.9 ± 0.3	7.2 ± 0.9	ND	ND

* p < 0.05 (high fat vs. chow fed); n = 9 for all groups

ND = not detectable; detection limit 5.6 pg/ml

### Diet-induced effects on plasma leptin, NPY and TNF-α

**Figure 2:**
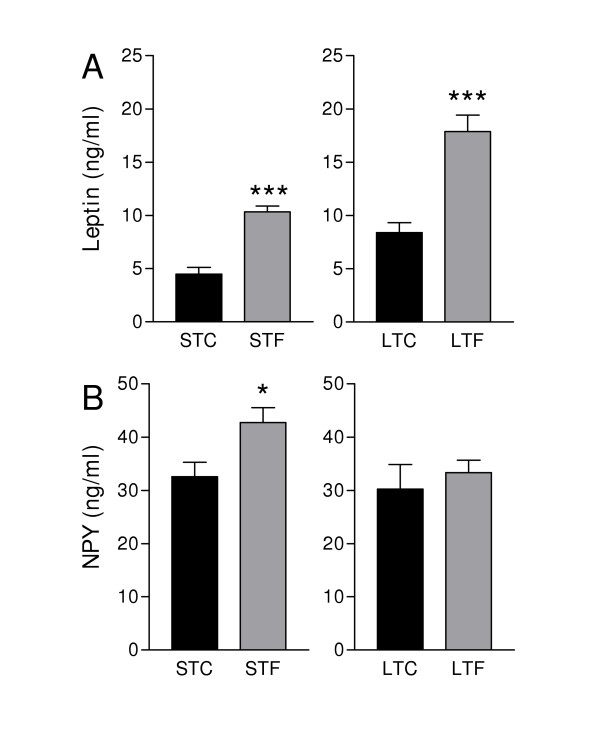
Plasma leptin (Fig. 2A) and NPY (Fig. 2B) concentrations after both types of dietary intervention: short term chow and fat diet for 2 weeks (STC/STF) and long term chow and fat diet (LTC/LTF). Results are expressed as mean ± SEM (n = 9 diet-induced obese rats, n = 9 control rats). Data were analysed by t-test: * p < 0.05, *** p < 0.0001.

Consumption of a high fat diet was associated with significant increases in plasma leptin concentrations (p < 0.05; Fig. 2). Even after 2 weeks on diet, leptin concentrations had more than doubled, at a time when body weight was not significantly elevated (Table 1). The chow fed rats also showed an increase in leptin concentrations from 2 to 10 weeks (Fig. 2), reflecting their increase in body weight and fat mass over time (Table 1). When plasma NPY concentrations were compared, consumption of the high-fat diet led to a significant increase (p < 0.05) in the short-term, whereas no change was observed after 10 weeks on diet (Fig. 2). There was no age-related change in plasma NPY concentrations in chow fed animals, indicating the absence of age-related effects on plasma NPY concentrations over this time period.

Despite other reports of increased plasma TNF-α concentrations in obesity [15], under the conditions used in this study, we failed to detect a significant difference between the chow and high-fat fed animals. In the older age group TNF-α levels were below the detection limit of the assay.

### LPS induced TNF-α production in full blood preparations

**Figure 3:**
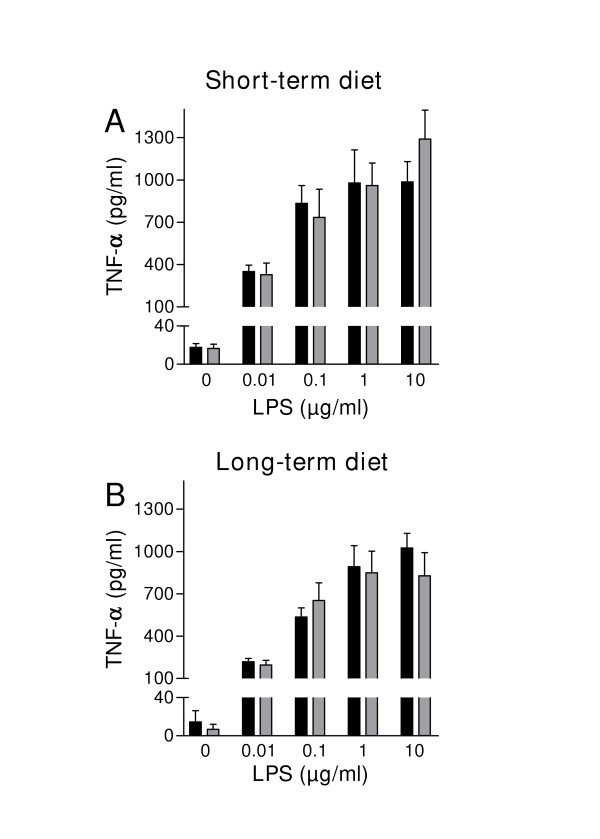
Stimulation of full blood preparations obtained from high-fat fed rats (grey bars) and control animals (black bars) after 2 weeks (short term) and 10 weeks on diet (long term) with increasing concentrations of LPS. Results are expressed as mean ± SEM (n = 9 diet-induced obese rats, n = 9 control rats).

To examine whether the high-fat diet modulates the ability of blood monocytes to produce TNF-α in response to LPS, full blood preparations from both chow and high-fat fed animals were compared. *Ex vivo *LPS-stimulation of full blood preparations resulted in a dose-dependent increase in the production of TNF-α (Fig. 3). However, the response to LPS did not differ significantly between animals fed chow and the high-fat diet at both time points examined (short term and long term diet, Fig. 3A and 3B).

### Stimulation of alveolar macrophages with LPS

**Figure 4:**
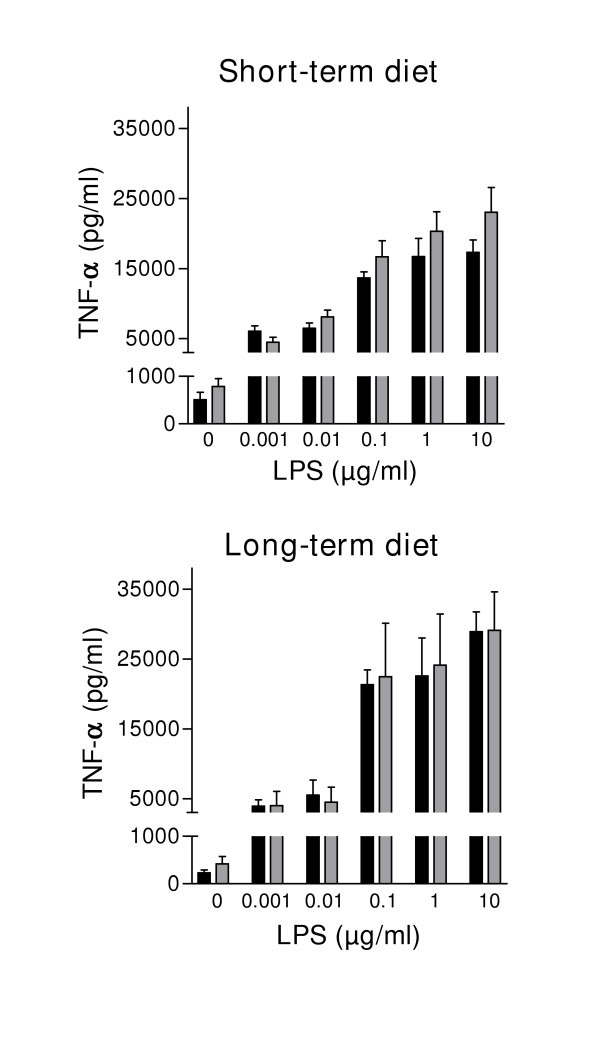
LPS stimulation of isolated alveolar macrophages after 2 weeks (short term) and 10 weeks on diet (long term) of high-fat fed rats (grey bars) and control animals (black bars). Results are expressed as mean ± SEM (n = 9 diet-induced obese rats, n = 9 control rats).

In order to examine whether the dietary intervention had any effect on functional parameters of tissue-borne macrophages, alveolar macrophages were stimulated with LPS *in vitro*. Increasing concentrations of LPS resulted in a dose-dependent, significant increase of the production of TNF-α by alveolar macrophages (Figure 4). There was no significant difference in the degree of stimulation by LPS in animals fed the high-fat diet for 2 or 10 weeks. Even though the basal production of TNF-α under these circumstances was not significantly different, high fat fed animals tended to have higher basal TNF-α levels, thus the proportional increase in the high-fat animals, expressed as percent change from basal, is suppressed in comparison to chow fed animals, particularly after long-term high fat feeding (13,747% versus 19,589% at 10 μg/ml LPS in fat and chow fed rats respectively).

## Discussion

We have previously extensively characterised the model of dietary obesity used in the current study [16,17]. Animals increase caloric intake on presentation of the diet, and show significant weight gain within 3 weeks. Reproducible increases in adiposity and plasma leptin concentrations occur within 2 weeks of high fat feeding, as demonstrated in the present study.

The majority of studies seeking to investigate the link between obesity and the immune system have carried out in genetic models of obesity. For example, defects in specific immunity, such as reduced lymphocyte numbers in spleen, thymus and the peripheral blood have been reported in *ob/ob *or *db/db *mice, and Zucker rats [2]. Furthermore, innate immune function seems also to be affected in genetic animal models of obesity. Specifically, it has been reported that macrophages from genetically obese animals have a reduced ability to eliminate Candida *albicans *and to produce proinflammatory cytokines [18]. While few studies have investigated immune function in diet-induced obesity, some changes in cellular and humoral immunity have been shown [19,20], however there is still no information on inflammatory immune function.

Leptin has also been demonstrated to modulate several functional immune parameters [6,8], and a recent study in humans demonstrated that leptin activates neutrophils indirectly by stimulating monocytes to release TNF-α[21]. We therefore asked whether diet-induced obesity, which is associated with significantly increased leptin levels and more closely resembles the most common form of human obesity than genetically modified models [22], would have an impact on innate immune functions. However, in the current study we found no alteration in the ability of macrophages and monocytes to release TNF-α to an LPS challenge.

We focused on blood monocytes and lung alveolar macrophages, as these cells are primary components of the innate branch of the immune system. Furthermore, with the current suggestion of a role for macrophages in driving the low-grade inflammation present in the adipose tissue [9], this approach also allowed us to evaluate whether intrinsic macrophage defects are present in obesity, as such defects would also occur in tissues other than the adipose tissue. Macrophages and monocytes produce TNF-α in response to innate immune stimuli such as LPS, which is essential for host defence against bacterial and other pathogens [23]. Our results demonstrate no obvious changes in the production of TNF-α by blood monocytes after 2 or 10 weeks of dietary intervention. It is possible that even though obesity had no influence on blood monocyte function, that the complex changes associated with obesity exert a functional influence on mature tissue-borne macrophages. However, using BAL-derived alveolar macrophages, we found no statistical difference in the net TNF-α response of alveolar macrophages upon LPS stimulation when comparing obese and control animals. Interestingly, when we analysed the percentile increase above basal TNF-α production at 10 weeks of diet, the high-fat fed animals appeared to have a blunted response to LPS, suggesting that alveolar macrophages from high-fat fed animals cannot be stimulated as strongly as the cells from the control animals.

More recently, obesity itself has been viewed as an inflammatory process [3,24,25] and studies in humans have demonstrated that weight loss can reduce inflammatory markers [26]. Thus recent attention has been focused on cytokines such as TNF-α and IL-6 [27]. TNF-α, formerly known as cachexin [28], has been studied in both animal models and human obesity. Some studies have shown that in humans increasing concentrations of leptin are correlated with soluble TNF-α receptors, suggesting the development of a pro-inflammatory state as body weight increases [29]. Adipose tissue itself is capable of producing TNF-α, and increased TNF-α concentrations in elderly subjects are correlated with truncal fat mass [30]. Several investigators have also reported increased plasma levels of TNF-α in genetic models of obesity. For example, plasma TNF-α concentrations were doubled in mice that were obese due to a defect in the growth hormone gene [15]. In our hands plasma TNF-α was not dramatically affected by the high fat diet, however this may be partly due to the fact that the values were very close to the detection limit of the assay. It is also probable that this discrepancy may highlight species differences, but it could also indicate that genetic obesity and diet-induced obesity impact differently on the regulation of TNF-α levels. Changes in TNF-α may be more consistent when there is a predominant genetic basis to the obesity where the level of obesity is usually more extreme [28]. Alteration in TNF-α may be tissue specific as shown by a recent study that proposes macrophage-related inflammatory activities in adipose tissue play a role in obesity-related insulin resistance [31].

Interestingly, we also found a significant increase in the concentration of circulating NPY after 2 weeks of dietary intervention. As peripheral NPY is predominantly derived from sympathetic nerve terminals, plasma NPY concentrations can be considered a marker of sympathetic nervous activity [13,14]. Thus, based on the present findings and previous reports of postprandial sympathetic activation [32], we propose that short term dietary excess increases sympathetic nervous activity. Increased sympathetic activity increases energy expenditure [33], and might therefore represent an endogenous mechanism to counteract weight gain. However, the question arises as to why plasma NPY levels are not different after 10 weeks of diet. Previous studies have shown that the changes in sympathetic nervous activity may be bed specific [34], with a higher renal and lower cardiac noradrenaline spillover in obese individuals [34]. Overall whole body sympathetic nervous activity in obese subjects was normal, which may explain why we do not see a change in plasma NPY levels after long-term diet exposure.

## Conclusion

Since specific gene defects only account for a small proportion of obesity in humans [7,35], this study was designed to investigate whether the functional defects in the monocyte/macrophage system described in genetically obese animal models are also present in an animal model of diet-induced obesity, that more closely resembles human obesity. The absence of any significant effects of diet-induced obesity on critical functional parameters of the monocyte/macrophage system used here raises the important question as to whether the changes in the production of pro-inflammatory cytokines observed in genetically obese animals actually result from the complex pathophysiology of obesity or are rather a consequence of the leptin defect present in these models. Our results favour the latter notion since our animals exhibit all the characteristics of obesity, yet do not display a comparable defect in the monocyte/macrophage system. Our results also show that monocyte and macrophage function in extra-adipose compartments is normal, suggesting that the chronic inflammatory state present in the adipose tissue during obesity is not a consequence of functional defects in the monocyte/macrophage system. One remaining possibility for our finding is that the period of overnutrition used does not reflect the changes observed in more chronic obesity.

While our results do not support a major effect of obesity on the markers of innate immune function used here, it does not rule out effects in other immune competent tissues, such as the endothelium. Clearly further work is required to delineate the possible effects of obesity on immune function, in light of the escalating burden of this disease.

## Competing interests

The authors declare that they have no competing interests.

## Authors' contributions

SaB participated in experimental design, carried out most experimental procedures and put together the manuscript. EV carried out the radioimmunoassays and helped to draft the manuscript. StB participated in sample collection and designed the LPS protocol. JEJ helped with sample collection. GPA participated in experimental design. MJM conceived of the study and participated in its design and coordination and helped to draft the manuscript. All authors read and approved the final manuscript.

## References

[B1] Jeanrenaud B, Rohner-Jeanrenaud F (2001). Effects of neuropeptides and leptin on nutrient partitioning: dysregulations in obesity. Annu Rev Med.

[B2] Marti A, Marcos A, Martinez JA (2001). Obesity and immune function relationships. Obes Rev.

[B3] Dandona P, Aljada A, Bandypadhyay A (2004). Inflammation: the link between insulin resistance, obesity and diabetes. Trends Immunol.

[B4] Stallone D (1994). The influence of obesity and its treatment on the immune system. Nutr Rev.

[B5] Loffreda S, Yang SQ, Lin HZ, Karp CL, Brengman ML, Wang DJ, Klein AS, Bulkley GB, Bao C, Noble PW, Lane MD, Diehl AM (1998). Leptin regulates proinflammatory immune responses. FASEB J.

[B6] Faggioni R, Fantuzzi G, Gabay C, Moser A, Dinarello CA, Feingold KR, Grunfeld C (1999). Leptin deficiency enhances sensitivity to endotoxin-induced lethality. Am J Physiol.

[B7] O'Rahilly S (2002). Leptin: defining its role in humans by the clinical study of genetic disorders. Nutr Rev.

[B8] Faggioni R, Feingold KR, Grunfeld C (2003). Leptin regulation of the immune response and the immunodeficiency of malnutrition. FASEB J.

[B9] Wellen EW, Hotamisligil GS (2003). Obesity-induced inflammatory changes in adipose tissue. J Clin Invest.

[B10] Bozinovski S, Jones JE, Vlahos R, Hamilton JA, Anderson GP (2002). Granulocyte/macrophage-colony-stimulating factor (GM-CSF) regulates lung innate immunity to lipopolysaccharide through Akt/Erk activation of NFkappa B and AP-1 in vivo. J Biol Chem.

[B11] Bedoui S, Miyake S, Lin Y, Miyamoto K, Oki S, Kawamura N, Beck-Sickinger S, von Hörsten S, Yamamura T (2003). Neuropeptide Y (NPY) suppresses experimental autoimmune encephalomyelitis: NPY1 receptor-specific inhibition of autoreactive Th1 responses in vivo. J Immunol.

[B12] Bedoui S, Pabst R, von Hörsten S, Michael, MC (2004). NPY and immune functions: implications for health and disease. Handbook of Pharmacology: NPY and related peptides.

[B13] Morris MJ, Russel AE, Kapoor V, Cain MD, Elliott JM, West MJ, Wing LM, Chalmers JP (1986). Increases in plasma neuropeptide Y concentrations during sympathetic activation in man. J Auton Nerv Syst.

[B14] Morris MJ, Kapoor V, Chalmers JP (1987). Plasma neuropeptide Y concentration is increased after hemorrhage in conscious rats: relative contributions of sympathetic nerves and the adrenal medulla. J Cardiovasc Pharmacol.

[B15] Ikeda A, Chang Kt, Matsumoto Y, Furuhata Y, Nishihara M, Sasaki F, Takahashi M (1998). Obesity and insulin resistance in human growth hormone transgenic rats. Endocrinology.

[B16] Hansen MJ, Ball MJ, Morris MJ (2001). Enhanced inhibitory feeding response to alpha-melanocyte stimulating hormone in the diet-induced obese rat. Brain Res.

[B17] Hansen MJ, Jovanovska V, Morris MJ (2004). Adaptive responses in hypothalamic neuropeptide Y in the face of prolonged high- fat feeding in the rat. J Neurochem.

[B18] Plotkin BJ, Paulson D, Chelich A, Jurak D, Cole J, Ksimos J, Burdick JR, Casteel N (1996). Immune responsiveness in a rat model for type II diabetes (Zucker rat, fa/fa): susceptibility to Candida albicans infection and leucocyte function. J Med Microbiol.

[B19] Lamas O, Martinez JA, Marti A (2004). Energy restriction restores the impaired immune response in overweight (cafeteria) rats. J Nutr Biochem.

[B20] Mito N, Kitada C, Hosoda T, Sato K (2002). Effect of diet-induced obesity on ovalbumin-specific immune response in a murine asthma model. Metabolism.

[B21] Zarkesh-Esfahani H, Pockley AG, Wu Z, Hellewell PG, Weetman AP, Ross RJ (2004). Leptin indirectly activates human neutrophils via induction of TNF-alpha. J Immunol.

[B22] Inui A (2003). Obesity – a chronic health problem in cloned mice?. Trends Pharmacol Sci.

[B23] Bochkov V, Kadl A, Huber J, Gruber F, Binder BR, Leitinger N (2002). Protective role of phospholipid oxidation products in endotoxin-induced tissue damage. Nature.

[B24] Grimble RF (2002). Inflammatory status and insulin resistance. Curr Opin Clin Nutr Metab Care.

[B25] Black P (2003). The inflammatory response is an integral part of the stress response: Implications for atherosclerosis, insulin resistance, type II diabetes and metabolic syndrome X. Brain Behav Immun.

[B26] Hukshorn CJ, Lindeman JH, Toet KH, Saris WH, Eilers PH, Westerterp-Plantenga MS, Kooistra T (2004). Leptin and the proinflammatory state associated with human obesity. J Clin Endocrinol Metab.

[B27] Chan JC, Cheung JC, Stehouwer CD, Emeis JJ, Tong PC, Ko GT, Yudkin JS (2002). The central roles of obesity-associated dyslipidaemia, endothelial activation and cytokines in the Metabolic Syndrome – an analysis by structural equation modelling. Int J Obes Relat Metab Disord.

[B28] Warne J (2003). Tumour necrosis factor alpha: a key regulator of adipose tissue mass. J Endocrinol.

[B29] van Dielen FM, van't Veer C, Schols AM, Soeters PB, Buurman WA, Greve JW (2001). Increased leptin concentrations correlate with increased concentrations of inflammatory markers in morbidly obese individuals. Int J Obes Relat Metab Disord.

[B30] Pedersen M, Bruunsgaard H, Weis N, Hendel HW, Andreassen BU, Eldrup E, Dela F, Pedersen BK (2003). Circulating levels of TNF-alpha and IL-6-relation to truncal fat mass and muscle mass in healthy elderly individuals and in patients with type-2 diabetes. Mech Ageing Dev.

[B31] Xu H, Barnes GT, Yang Q, Tan G, Yang D, Chou CJ, Sole J, Nichols A, Ross JS, Tartaglia LA, Chen H (2003). Chronic inflammation in fat plays a critical role in the development of obesity-related insulin resistance. J Clin Invest.

[B32] Tappy L, Girardet K, Schwaller N, Vollenweider L, Jequier E, Nicod P, Scherrer U (1995). Metabolic effects of an increase of sympathetic activity in healthy humans. Int J Obes Relat Metab Disord.

[B33] Kaufman LN, Young JB, Landsberg L (1986). Effect of protein on sympathetic nervous system activity in the rat. Evidence for nutrient-specific responses. J Clin Invest.

[B34] Rumantir MS, Vaz M, Jennings GL, Collier G, Kaye DM, Seals DR, Wiesner GH, Brunner-La Rocca HP, Esler MD (1999). Neural mechanisms in human obesity-related hypertension. J Hypertens.

[B35] Bray G, Bouchard C (1997). Genetics of human obesity: research directions. FASEB J.

